# Multi-Center Study on Sample Preparation and Feature Selection Effects in Reproducible Mass Spectrometry Imaging for Clinical Translation

**DOI:** 10.1016/j.mcpro.2026.101658

**Published:** 2026-09-10

**Authors:** Juliana P.L. Gonçalves, Marta Grzeski, Gentiane Krasniqi, Carsten Hopf, Oliver Klein, Kristina Schwamborn

**Affiliations:** 1 Technical University of Munich, TUM School of Medicine and Health, Institute of Pathology, Munich, Germany; 2 Imaging Mass Spectrometry Unit, Berlin Institute of Health (BIH) at Charité, Berlin, Germany; 3 Center for Mass Spectrometry and Optical Spectroscopy (CeMOS), Technische Hochschule Mannheim, Mannheim, Germany; 4 CeMOS Research and Transfer Center, Mass Spectrometry and Optical Spectroscopy, Technical University Mannheim, Mannheim, Germany; 5 Mannheim Center for Translational Neuroscience (MCTN), Medical Faculty Mannheim, Heidelberg University, Mannheim, Germany; 6 Medical Faculty Heidelberg, Heidelberg University, Heidelberg, Germany

**Keywords:** Mass spectrometry imaging, In situ proteomics, multi-center study, clinical translation, MALDI-MSI

## Abstract

Matrix-assisted laser desorption/ionization mass spectrometry imaging (MALDI-MSI) provides spatially resolved proteomic information that is valuable for understanding disease development, identifying biomarkers, and predicting treatment responses. Despite this potential, MALDI-MSI has not yet been adopted as part of routine diagnostic workflows, primarily due to challenges in analytical validation and robustness. Although sample preparation workflows for MALDI-MSI generally follow the same core steps, there is still a lack of robust data-processing frameworks capable of minimizing technical artifacts, improving feature selection, and reducing inter-laboratory variability. To assess reproducibility and robustness of *in situ* proteomics across laboratories, we investigated formalin-fixed paraffin-embedded human tissue samples from spleen, intestine, and pancreas using MALDI-MSI. Two complementary workflows were evaluated: (*i*) decentralized sample preparation with data acquisition performed with one instrument and (*ii*) centralized sample preparation with data acquisition carried out on instruments across participating laboratories. Data analysis focused on evaluating strategies for feature selection that preserved morphological information. Our findings demonstrate that, with adherence to a standardized protocol, reproducible results across different laboratories can be achieved. Eventhough sample preparation remains a major source of variability, it can be mitigated through appropriate data analysis strategies, particularly in the choice of features used for analysis. Most importantly, biological differences between tissues were consistently identifiable across all datasets, further underscoring the reliability of the approach for clinical translation, especially when sources of variability are properly controlled.

## Highlights

• Multicenter MALDI-MSI shows tissue biology outweighs technical variability.
• Sample preparation, not instrumentation, drives most inter-laboratory variability.
• Feature selection spanning a broad mass range outperforms intensity-only approaches.
• Standardized preprocessing and transparent workflows ensure reproducible MSI studies.

## Graphical Abstract

**Figure:**
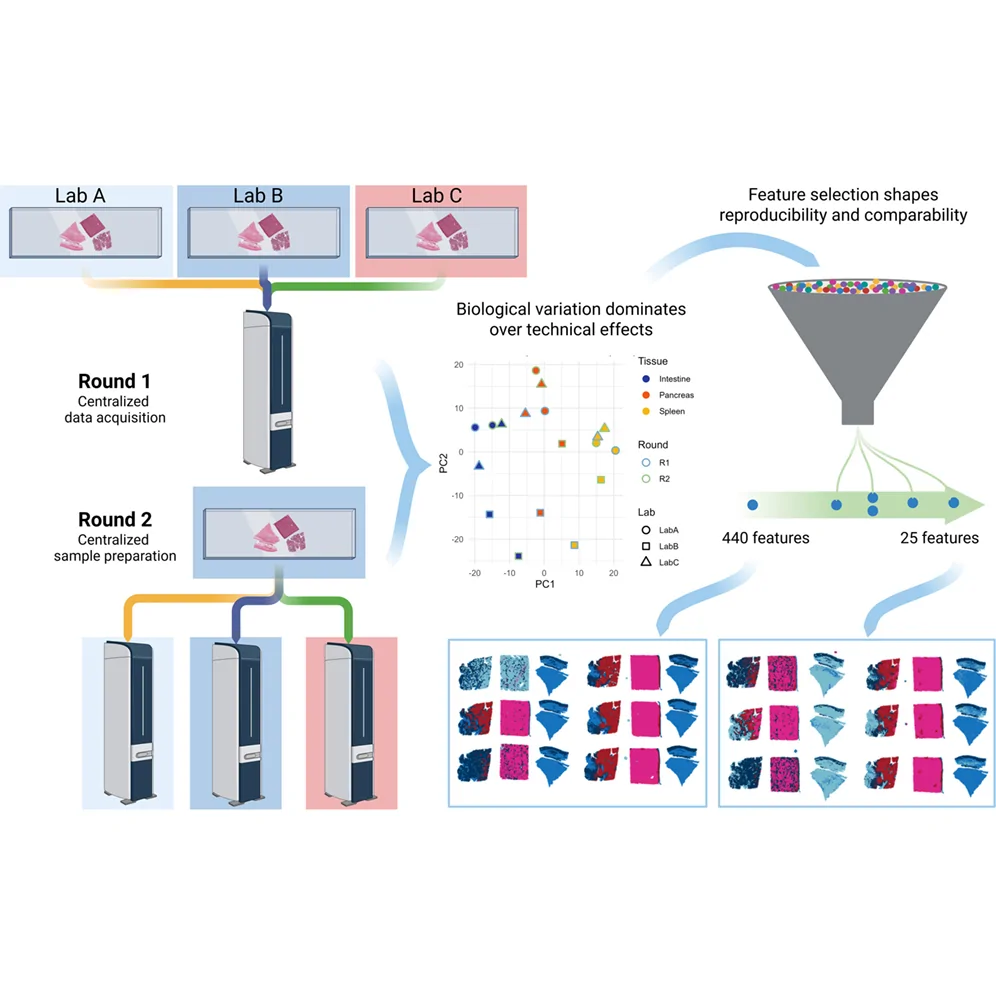

## In Brief

MALDI mass spectrometry imaging has great potential for clinical tissue analysis but requires robust and reproducible workflows for routine use. In this multicenter study, we show that standardized protocols produce consistent biological results across laboratories. We identify sample preparation as the main source of technical variability and demonstrate that optimized feature selection substantially improves reproducibility. These findings provide a practical framework for increasing the reliability of MALDI-MSI and accelerating its clinical translation.

Matrix-assisted laser desorption/ionization mass spectrometry imaging (MALDI-MSI) has emerged as a transformative technique in the field of molecular analysis, providing detailed molecular information while preserving the spatial localization of analytes (1). This powerful approach has found numerous applications, particularly in biomedical research such as pharmacology, biomarker discovery, and tissue/disease classification (2, 3, 4, 5, 6, 7, 8, 9). Despite the growing body of research demonstrating its utility for clinical translation, comprehensive evaluations of its performance, in particular with regards to interlaboratory reproducibility, remain limited. Additionally, there is no consensus on data analysis strategies that would enable standardized processing and ensure a biologically meaningful interpretation (10).

One of the key advantages of MALDI-MSI is that it is a label-free technique, enabling the simultaneous analysis of multiple compounds in a single run while maintaining the spatial context crucial for correlating molecular data with histological features in a complex microenvironment. This allows researchers to leverage spatial molecular information to differentiate tissue regions and identify specific molecular characteristics associated with tissue compartments, cell types, and pathological conditions (11, 12, 13, 14, 15, 16). Since MALDI-MSI workflows are compatible with formalin-fixed paraffin-embedded (FFPE) tissue samples, the gold standard in anatomical pathology, and can be seamlessly aligned with histopathological processes, the technology holds the promise to become a powerful tool in day-to-day pathology practice. Despite its numerous advantages, the effective use of MALDI-MSI to support diagnostic decision-making requires comprehensive evaluations of reproducibility and comparability of results within a single lab over time as well as across different labs. Instrumental reliability and performance, robust and reproducible sample preparation, as well as data processing consistency across institutions, have to be carefully evaluated (17).

To address these challenges, multi-institutional studies on the reproducibility of MALDI-MSI of tryptic peptides have been conducted (18, 19). The study by Buck *et al*. compared the inter- and intracenter variability of peptide and metabolite profiles across four laboratories. Inter-center variation for peptides was significantly lower than intra-center variation, and multivariate classifiers trained on sufficiently diverse cross-center data achieved average accuracies exceeding 90% for peptides (18). Another study, performed by Ly *et al*., demonstrated that both instrumentation parameters (*e.g*., laser diameter) and sample preparation factors (*e.g*., matrix and trypsin application) strongly influence data quality (19). However, when sample preparation and measurement protocols are carefully harmonized in a standard operating procedure, MALDI–MSI can yield reproducible results across sites. The influence of technical variability and data processing on cross-site reproducibility and classifier transferability for MALDI-MSI-based tissue phenotyping was evaluated by Deininger *et al*. (20) Protocol harmonization and optimized data preprocessing improved classification accuracy substantially. Another study by Fagerer *et al*. aimed to establish a standardized framework for MALDI-MSI data processing and demonstrated that standardized sample preparation is essential for achieving consistent results across locations (21). Taken together, these studies all highlight two key insights: (1) technical variability, such as differences in sample preparation, significantly influence inter-site data comparability and classifier generalizability, and (2) preprocessing workflows can effectively mitigate site-specific differences.

While previous studies have demonstrated a high degree of inter-laboratory reproducibility in molecular profiling, a certain level of variability persists, even when standardized sample preparation protocols are followed and adjacent tissue sections are analyzed (17, 21, 22). In addition to analytical considerations, external factors affecting sample preparation, such as environmental conditions (*e.g*., room temperature and humidity), and variability introduced during tissue fixation and embedding, can also contribute to inconsistency (23, 24, 25). Controlling these parameters within a single institution is already challenging, and achieving uniform preanalytical conditions in a clinical setting is nearly impossible. Ensuring consistency across multiple sites introduces additional obstacles and complexity, underscoring the persistent difficulty of obtaining fully reproducible results across laboratories and the need for effective strategies to address these issues (26, 27).

Importantly, sources of variability can both originate from and be mitigated by data processing and analysis, including preprocessing workflows and normalization strategies (28). Therefore, while the standardization of sample preparation protocols remains essential, it must be complemented by robust data processing frameworks that minimize technical differences and maximize the extraction of biologically meaningful signals. Among these steps, feature selection plays a critical role in shaping analytical outcomes, yet it has not been comprehensively addressed in previous multicenter efforts (10, 29).

To bridge this gap, our study systematically evaluated technical reproducibility, instrumental robustness, and the impact of feature selection strategies on data interpretation and reproducibility. We applied a harmonized sample preparation protocol across three independent laboratories to analyze human tissue specimens (*i.e*., spleen, intestine, and pancreas) and employed different feature selection approaches for the analysis. This multicenter study aims to identify key contributors to variability in MSI-based proteomic studies and provide recommendations to improve reproducibility, particularly in translational and clinical research settings.

## Experimental Design and Statistical Rationale

This study was designed as a multicenter reproducibility assessment rather than a biomarker discovery experiment. To minimize biological variability and enable direct evaluation of technical sources of variation, three human tissue types (intestine, pancreas, and spleen) were embedded within a single FFPE block and analyzed as consecutive tissue sections. This design allowed biological composition to remain as constant as possible while systematically varying experimental conditions.

The study comprised three complementary experimental rounds. In Round 1, tissue preparation was performed independently at three participating laboratories, followed by centralized MALDI-MSI acquisition, enabling assessment of variability arising from sample preparation. In Round 2, tissue preparation was centralized and data acquisition was performed independently at the participating laboratories, enabling assessment of instrument- and acquisition-related variability. Round 3 served as a quality-control experiment to evaluate intra-laboratory reproducibility and the potential effects of sample storage. In total, six slides were analyzed for inter-laboratory comparisons and four slides for intra-laboratory assessment, corresponding to 24 tissue sections across all experiments. The use of consecutive tissue sections from the same donor material was intentional and allowed technical variability to be evaluated while minimizing confounding biological differences.

The primary experimental unit for statistical analysis was the independently prepared and/or acquired tissue section rather than individual MSI pixels, as pixels within a tissue section are not statistically independent. Mean feature intensities extracted from annotated tissue regions were therefore used for downstream statistical analyses, and reproducibility was assessed across independently processed slides, laboratories, instruments, and experimental rounds.

Statistical analyses were designed to quantify analytical reproducibility and identify sources of technical variation rather than to test for differential abundance between biological groups. Reproducibility between experimental rounds was evaluated using pairwise correlation analyses and Lin's concordance correlation coefficient. Principal component analysis was used to visualize the relative contributions of tissue type, laboratory, and experimental round to the overall variance. In addition, feature-wise analysis of variance models were fitted to partition variance attributable to tissue type, laboratory, and experimental round, with effect sizes quantified using eta-squared (η^2^).

## Experimental procedures

The sample preparation workflow (Fig. 1*A*) was utilized across the whole study which was conducted in three experimental rounds (Fig. 1*B*). In Round 1, three slides were processed individually in different laboratories but measured in a single laboratory using the same mass spectrometer. In Round 2, all slides were processed in one laboratory and measured separately in each laboratory using their respective mass spectrometers. In Round 3, intra-lab variability was evaluated by preparing two slides in one lab and measuring one sample on the same day of sample preparation, and the other samples on synchronized days with the other participating labs. This was done during both experimental Rounds 1 and 2. Round 3 experiment also aimed at identifying potential effects related to sample storage after sample preparation, required for the shipping process.

**Fig. 1:**
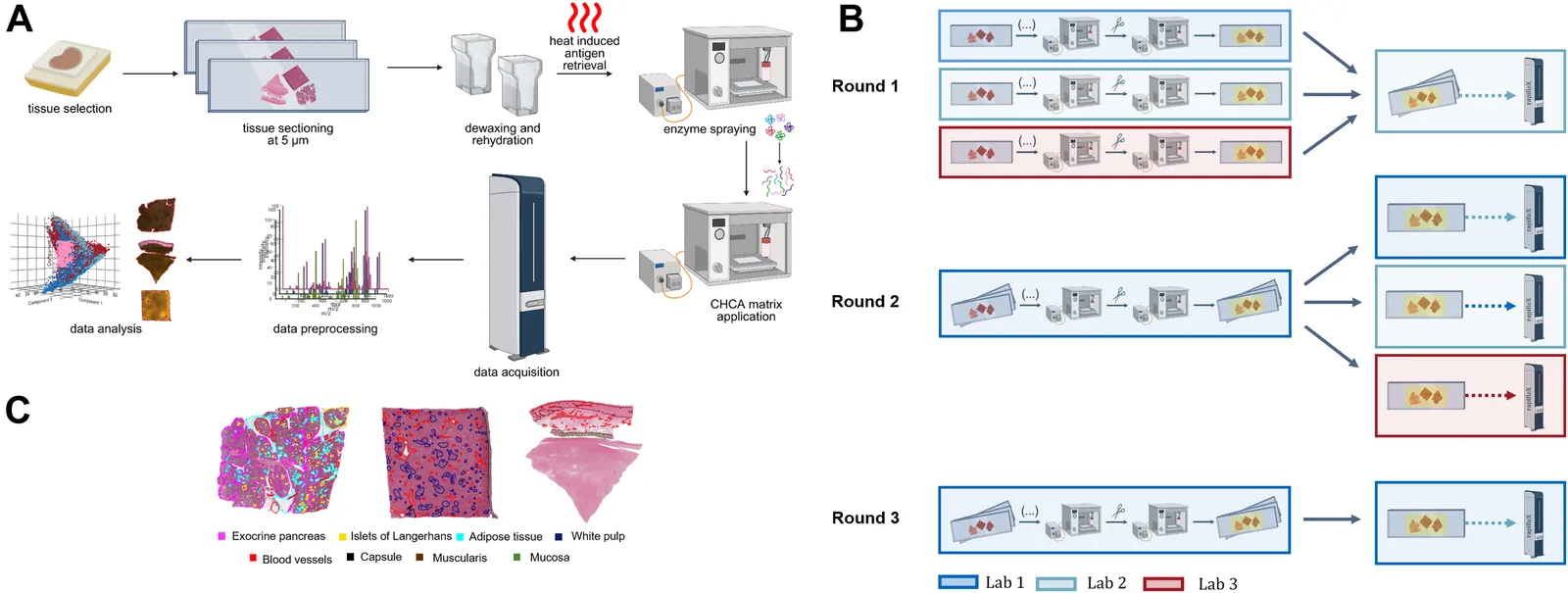
**Expetimentaldesign.** (*A)* experimental workflow; (*B*) schematic representation of the different experimental rounds. Round 1: decentralized sample preparation with centralized acquisition. Round 2: centralized sample preparation with decentralized acquisition. Round 3: quality-control assessment of storage/shipping effects. *C*, hematoxylin and eosin-stained tissue section with histological annotations of the main compartments of the tissues included in the study. The annotations were performed in QuPath. This figure was partially created in BioRender. Pereira Lopes Goncalves, J. (2026) https://BioRender.com/n7ylb69.

### Tissue Samples

Surgical resections of human intestine, spleen, and pancreas were collected at the Institute of Pathology, Technical University of Munich and formalin-fixed paraffin-embedded in the same block. Tissue fixation and embedding were performed according to a standardized, automated protocol used in routine pathology. Briefly, tissue samples were fixed in 4% neutral buffered formalin for 12 to 24 h, dehydrated in a graded series of ethanol, cleared with xylene, and embedded in molten paraffin. Serial sections, 5 μm thick, were mounted onto indium-tin-oxide (ITO)-coated glass slides (Bruker Daltonics) pretreated with a 1:10 aqueous mixture of poly-ʟ-lysine (Sigma-Aldrich GmbH). The slides were dried overnight at 37 °C and either distributed across all three participating laboratories (Charité – Universitätsmedizin Berlin (BIH), Center for Mass Spectrometry and Optical Spectroscopy (CeMOS), and Technical University of Munich (TUM)) for the Round 1 experiment, or stored in a drying chamber until further processing (TUM) in Rounds 2 and 3. Shipment and storage prior to the sample preparation and data acquisition were conducted at ambient and room temperature, respectively. The study was conducted in accordance with the Declaration of Helsinki and approved by the institutional ethics review board (Ethics Committee of the School of Medicine and Health, Technical University of Munich, Protocol Number 16/22S).

### Tissue Processing

Tissue processing was synchronized across labs and initiated on the same day according to the protocol by Ly *et al.* (19) with some modifications (Supplemental Table S1). The chemicals used in each laboratory are listed in Supplemental Table S2. The slides were incubated at 85 °C for 15 min, followed by deparaffinization in xylene (3 × 5 min), isopropanol (100%; 1 × 5 min), and rehydration through sequential ethanol washes (100%, 95%, 70%, and 50%; 1 × 5 min each), and MilliQ water (1 × 5 min). To reverse formaldehyde-induced protein cross-linking, heat-induced epitope retrieval was performed in deionized water following the established procedure of each laboratory. Epitope retrieval was carried out at 98 °C for 20 min in a steamer (FS10, BRAUN; BIH) or at 110 °C for 20 min using a decloaking chamber (Biocare Medical, Pacheco; TUM and CeMOS). Afterward, the slides were brought to room temperature by Milli-Q water exchanges, then dried in a desiccator for at least 25 min, until fully dried. The slides were scanned at 1200 dpi using slide scanners (MF5000 KLB, Reflecta GmbH, TUM and BIH; Aperio CS2, Leica Biosystems, CeMOS). Sequencing-grade modified trypsin (Promega) was reconstituted to a final concentration of 25 μg/ml in 20 mM NH_4_HCO_3_ (Sigma-Aldrich), supplemented with 0.01% glycerol, and evenly applied onto the tissue sections using an automated spraying device (HTX TM-sprayer, HTX Technologies). The spraying parameters were set as follows: 16 layers, flow rate of 0.015 ml/min, spray velocity of 750 mm/min, track spacing of 2 mm, and nozzle temperature set to 30 °C. The enzymatic digestion was carried out in a digestion chamber containing a saturated K_2_SO_4_ solution to maintain a stable humidity of 96%. The chamber was placed in an oven pre-heated to 50 °C and incubated for 2 h. After digestion, the samples were sprayed with α-cyano-4-hydroxycinnamic acid (CHCA; Bruker Daltonics GmbH) matrix solution (7 mg/ml) in 70% ACN and 1% TFA using the following parameters: four layers, flow rate of 0.120 ml/min, spray velocity of 1200 mm/min, track spacing of 3 mm, and nozzle temperature of 75 °C. Following matrix application, peptide calibration standard II (0.5 μl; Bruker Daltonics GmbH) was manually spotted in a tissue-free area of each slide. The prepared slides were either shipped at ambient temperature to another laboratory for analysis or stored at room temperature for subsequent measurement in the same laboratory. All measurements, whether performed locally or externally, were conducted on a synchronized day to ensure consistency in data collection.

### MALDI-TOF-MS Measurement

All analyses were performed using RapifleX MALDI-TOF Tissuetyper mass spectrometers (Bruker Daltonics GmbH), equipped with Smartbeam 3D Nd lasers (355 nm), operating in positive ionization reflector mode. Mass spectra were collected over a mass-to-charge (*m/z*) range of 600 to 3200, with a lateral spatial resolution of 50 × 50 μm. Each spectrum was generated using 500 laser shots per spot at a sampling rate of 1.25 GS/s, with baseline subtraction applied during acquisition (acquisition method details for each acquisition are detailed in Supplemental Table S3). Laser power was adjusted at each measurement location to optimize ionization efficiency. For quality control, a non-tissue region was included in each measurement as a reference. External calibration was performed using the Peptide Calibration Standard II (PepMix, Bruker Daltonics GmbH) (Supplemental Fig. S1). MSI data were acquired using flexImaging (v. 5.1 or 6.0) and flexControl (v. 3.0 or 4.2) software (Bruker Daltonics GmbH). Following data acquisition, the CHCA matrix was removed from a couple of slide samples with 70% ethanol (2 × 2 min) and MilliQ water (1 × 1 min). Two slides were then stained with hematoxylin and eosin (H&E) and scanned at 40 × magnification using an Aperio AT2 DX System slide scanner (Leica Biosystems) and histological annotations (Fig. 1*C*) were performed by a board-certified pathologist.

### Data Preprocessing

MALDI-MSI raw data of each experimental round were imported into SCiLS Lab 2024a Pro (Bruker Daltonics GmbH) software. The generated MALDI-TOF-MS datasets were then aligned based on the chemical noise background with the help of SCilS_align_peptides tool (v 0.1.0) (17, 30). The aligned files were reimported into SCiLS Lab software and used for further analyses. The pre-processing of mass spectra included baseline removal using TopHat algorithm with peak width set to 200 and total ion count (TIC) normalization. Peak finding was conducted using a standard segmentation pipeline, implementing TIC normalization, no denoising, and minimal interval width of ±0.128 Da (17, 30, 31, 32, 33). Intensity of each detected feature was computed based on its average intensity in a respective region, selecting peak maximum as interval processing mode.

### Data Analysis

For the data analysis, we generated six distinct feature lists: (1) a comprehensive list including all detected features in the *m/z* range 600 to 3200, resulting in 440 *m/z* values; (2) a focused list containing all features within the *m/z* range 1150 to 2250, totalizing 226 features; (3) a list of the 100 most intense features across the entire spectrum; (4) a list with the 100 most intense monoisotopic features; (5) a curated list of the top 25 features selected from defined mass intervals, designed to ensure broader, yet proportional, representation across the full *m/z* range (Supplemental Fig. S2 and Supplemental Table S4); and (6) a list with the 50 most intense features from a spectrum generated by a region in the principal component analysis (PCA) where the different measurements intersected (Supplemental Fig. S3 and Supplemental Table S4). The PCA was performed using five components, no denoising, a peak maximum interval processing mode, unit variance scaling, and a skyline spectrum mode. For k-means clustering, the analysis was performed with five clusters, no denoising, a peak maximum interval processing mode, correlation distance as the metric, and “all spectra” as the spectrum mode.

A matrix of the average intensity per feature, per tissue, including the round information and the laboratory of sample preparation or data acquisition was exported to R (version R-4.0.4), utilizing R Studio (version 2025.05.0 + 496 "Mariposa Orchid" Release, 2025-05-04) as environment, for statistical analysis. Aside from the base R functions, the “tidyverse”, “dplyr”, “ggplot2”, “stringr”, “purrr”, “corrplot”, “UpSetR”, “patchwork”, and dependent packages were loaded into the environment. For all feature lists, we have compared the intensity of the features across the different labs and the different rounds. Additionally, feature lists include a pairwise correlation between the different measurements and changes in intensity per lab and round.

To assess inter-laboratory variability, we applied a regression-based correction to remove lab-specific biases while preserving biological signals. For each consensus feature (defined by *m*/*z* clustering), log-transformed intensities (log_10_(1 + intensity)) were modeled using linear regression: Intensity ∼ Tissue + Round + Lab, with Lab included as a fixed effect to account for systematic differences between experiments. Corrected intensities were computed as the predicted values from the reduced model (Tissue + Round) plus the residuals from the full model, effectively removing lab-specific offsets while retaining biological variation. Corrections were applied feature-wise, and no scaling was performed during PCA to preserve the original magnitude of biological variation.

Agreement between measurement rounds (R1 and R2) was assessed using Lin’s concordance correlation coefficient (CCC). Mean intensities were first computed per laboratory and round, and only paired observations with non-missing values were retained. CCC was calculated using the DescTools package in R, with a custom wrapper to ensure robustness to output format or calculation errors; in such cases, Pearson correlation (pairwise complete observations) was used as a fallback. CCC was only computed when at least two paired observations were available.

For each dataset and each *m/z* feature, signal intensity was modeled using a fixed-effects, multi-factor ANOVA with additive main effects: Intensity ∼ Round + Lab + -Tissue. Effect sizes were quantified as eta-squared (η^2^) for each factor (round, lab, tissue) using sequential (Type I) sums of squares in the stated order (round, lab, tissue). Specifically, η^2^ was computed from the ANOVA table as η^2^ = SSterm/SStotal (DescTools::EtaSq, type = 1). To summarize the distribution of effect sizes across features, kernel density estimates of η^2^ were plotted per factor and faceted by dataset, enabling comparison of the relative contributions of round, lab, and tissue to average intensity variability. Further representations of the PCA using the average intensities were plotted. Long-format intensities were reshaped into a samples-by-features matrix; sample metadata (round, lab, tissue) were parsed from sample identifiers and joined to the scores. PCA was run on centered (mean-subtracted), non-scaled features using prcomp. The variance explained by PC1 and PC2 was reported in the axis labels. For visualization, PC1–PC2 score plots were rendered in two panels per dataset: one colored by lab and one by round (Supplemental Fig. S4).

### Protein Identification by Nano-LC-ESI-MS/MS

A consecutive tissue section was used for bottom-up nano-liquid chromatography electrospray ionization tandem mass spectrometry (nano-LC-ESI-MS/MS) to identify *m/z* features detected by MALDI-MSI. The slides were processed following the same protocol as described above for MALDI-MSI up to enzymatic digestion. The generated peptides were extracted from each tissues’ surface with 50 μl of 0.1% TFA at room temperature, and desalted with ZipTip C18 pipette tips (Merck KGaA) following the manufacturer’s protocol. After drying under vacuum centrifugation, eluates were reconstituted in 20 μl of 0.1% TFA and stored at −20 °C until analysis. For nano-LC-ESI-MS/MS analysis, 6 μl of each eluted fraction were injected into a Dionex Ultimate 3000 nano-HPLC system (Thermo Fisher Scientific) coupled to a timsTOF HT fleX mass spectrometer (Bruker Daltonics GmbH) equipped with a CaptiveSpray ESI source. Peptides were loaded in 0.1% TFA onto an Acclaim PepMap 100 C18 trap column (2 cm × 100 μm × 5 μm, Thermo Fisher Scientific) and separated on an Aurora ultimate CSI C18 analytical column (25 cm × 75 μm × 1.7 μm; Bruker Daltonics GmbH) using a 60-min gradient from 3% to 37% acetonitrile in 0.1% formic acid. The flow rate was 400 nl/min, the column temperature 60 °C, and the system operated within a pressure range of 10 to 800 bar.

Mass and ion mobility calibration were conducted using the ESI-LC tuning mix (Agilent Technologies, Inc), employing enhanced quadratic and linear calibration modes, respectively. Data were acquired using the DDA-PASEF method with TIMS enabled. The mass spectrometer operated over 100 to 1700 *m/z* range, with an ion mobility window of 0.85 to 1.30 Vs/cm^2^ and a TIMS ramp time of 100 ms. Precursor ions were fragmented via collision-induced dissociation. Raw data (.baf) were analyzed using PEAKS Studio 12 (Bioinformatics Solutions Inc., Waterloo, Canada) (34) searching against the UniProt human database (*Homo sapiens* taxonomy, downloaded on 31. October, 2023). 20,423 entries were searched, and parameters included a precursor mass tolerance of 20 ppm, 0.05 Da fragment mass tolerance, trypsin digestion (allowing up to two missed cleavages), and up to two variable modifications (methionine oxidation and *N*-terminal acetylation). A 1% FDR threshold was applied, with protein identification requiring at least one unique peptide. Only the top-scoring ID per protein group was retained.

## Results

In the first round (Round 1), tissue preparation was performed independently in each laboratory following the same standardized protocol. Afterward, the prepared samples were sent to a single laboratory (BIH) for data acquisition. In the second round (Round 2), tissue sections were sectioned and uniformly processed following the same protocol in a single laboratory (TUM) before being shipped to the respective locations for data acquisition. Additionally, our study included the analysis of three further tissue slides (designated as Round 3, Fig. 1*B*), for which the entire workflow, encompassing both sample processing and data acquisition, was conducted within a single laboratory (TUM). This study design enabled us to assess both inter- and intra-laboratory reproducibility and to identify the extent of variance arising from tissue processing and data acquisition steps. For inter-lab reproducibility, the final dataset consisted of six consecutive slides, each containing three human tissue specimens of pancreas, intestine, and spleen. For intra-lab reproducibility assessment, four slides were considered, each also containing three tissue sections from the same tissues, as in Rounds 1 and 2. In total, 24 tissue sections were analyzed. The average mass spectra for each tissue specimen, acquired in experimental Rounds 1 and 2, are presented in Figure 2*A* and more detailed in Supplemental Figs. S5–S7.

**Fig. 2:**
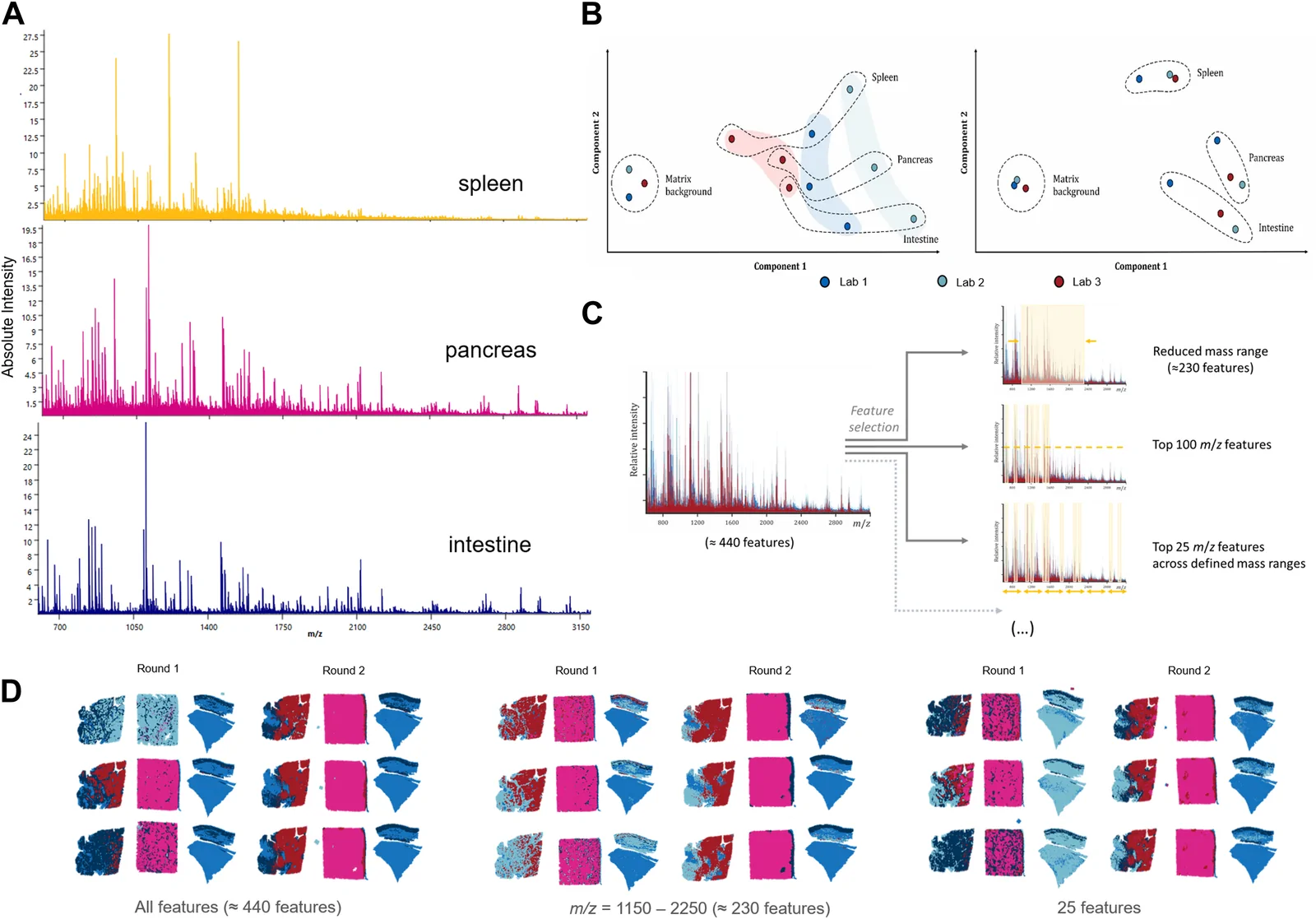
**Biological differences dominate over round and lab effects, and feature selection improves agreement.***A*, average spectra per tissue type included in the study (spleen, pancreas, and intestine) across all sites for Rounds 1 (centralized data acquisition) and 2 (centralized sample preparation). *B*, PCA of datasets acquired during experimental Rounds 1 and 2 using all detected features. Dashed outlines were manually added to indicate the different measurement regions, and colored backgrounds were manually added to visually emphasize measurement regions acquired in the same laboratory. *C*, feature reduction approaches. *D*, unsupervised k-means clustering analysis of datasets recorded in Rounds 1 and 2 employing different feature selection approaches.

### Using all Features Enables Discrimination of Tissue Types and Morphological Regions But Also Reveals Inter-round Variability

To evaluate the similarities between peptide profiles recorded from samples processed at different sites (Round 1) and those processed in one lab but measured at different sites (Round 2), PCA was performed on data from all tissues. Additionally, to assess the ability of the acquired data to discriminate biological variability across tissue sections tested in all labs, k-means clustering with five clusters was employed (Fig. 2*D*). These unsupervised analyses considered all features obtained by the “feature finding” function in SCiLS lab, totalizing 440 features. Notably, in Round 2 dataset, PCA and k-means clustering patterns, predominantly reflected the biological characteristics of the investigated tissues (Fig. 2). The same trend was overall also observed in Round 1, however clustering patterns somewhat also reflected sample processing effects aside from the biological differences. This is especially visible in the pancreas and spleen tissues.

Given that sample preparation effects are expected and can be minimized, but not entirely eliminated, we sought to evaluate whether such variability could be further reduced through data analysis, specifically via feature selection (28, 35). As feature selection plays a critical role in proteomic data interpretation and directly influences analytical outcomes, we assessed how different selection strategies impact the dataset, particularly in the context of spatially resolved approaches where standardized guidelines are lacking and reporting is often inconsistent (36, 37, 38).

### Feature Selection Improves Interlaboratory Comparability

We aimed to assess whether selected subsets of features enhance the capture of biological information and inter-lab comparability compared to using the entire dataset. Five feature selection strategies were evaluated and compared to unsupervised analysis using all detected features (Table 1). f226 mass range reduction (*m/z* 1150–2250); f100 the top 100 *m/z* features across the entire mass range (*m/z* 600–3200); f100m the top 100 *m/z* features across the entire mass range (m/z 600–3200), manually peaked to include only (to the best of our knowledge) monoisotopic peaks; f25 the top 25 *m/z* features across the entire mass range, selected to reflect the percentage distribution of all detected features, and; f50 the top 50 peaks selected from the average spectra of a region where all the different clusters intersected in a PCA. The overlap in the features across the different feature selection approaches is represented in Figure 3.

**Table 1:** Summary of the Feature Selection Approaches

Approach	Description	Characteristics
f440	Full dataset (440 *m/z* features)	*m/z* 600–3200 Contains all *m/z* features identified by the “feature finding” function in SCiLS Lab
f226	Mass range reduction (226 *m/z* features)	*m/z* 1150–2250
f100	Top 100 *m/z* features across the entire mass range, selected by intensity	*m/z* 600–3200
f100m	Top 100 *m/z* features across the entire mass range, selected manually to include only monoisotopic peaks	Potentially includes a higher number of different analytes than the other feature selection approaches; requires skill and is time-consuming
f25	Top 25 *m/z* features across the entire mass range, selected to reflect the percentage distribution of all detected features	Reflects the relative abundance distribution of all detected features; time-consuming
f50	Top 50 peaks selected from the average spectra of a region where all clusters intersect in PCA	Region corresponding to the overlap of all clusters identified by principal component analysis (PCA; see Supplemental Fig. S3 for the graphical representation); this approach does not represent the totality of the different tissue regions

**Fig. 3:**
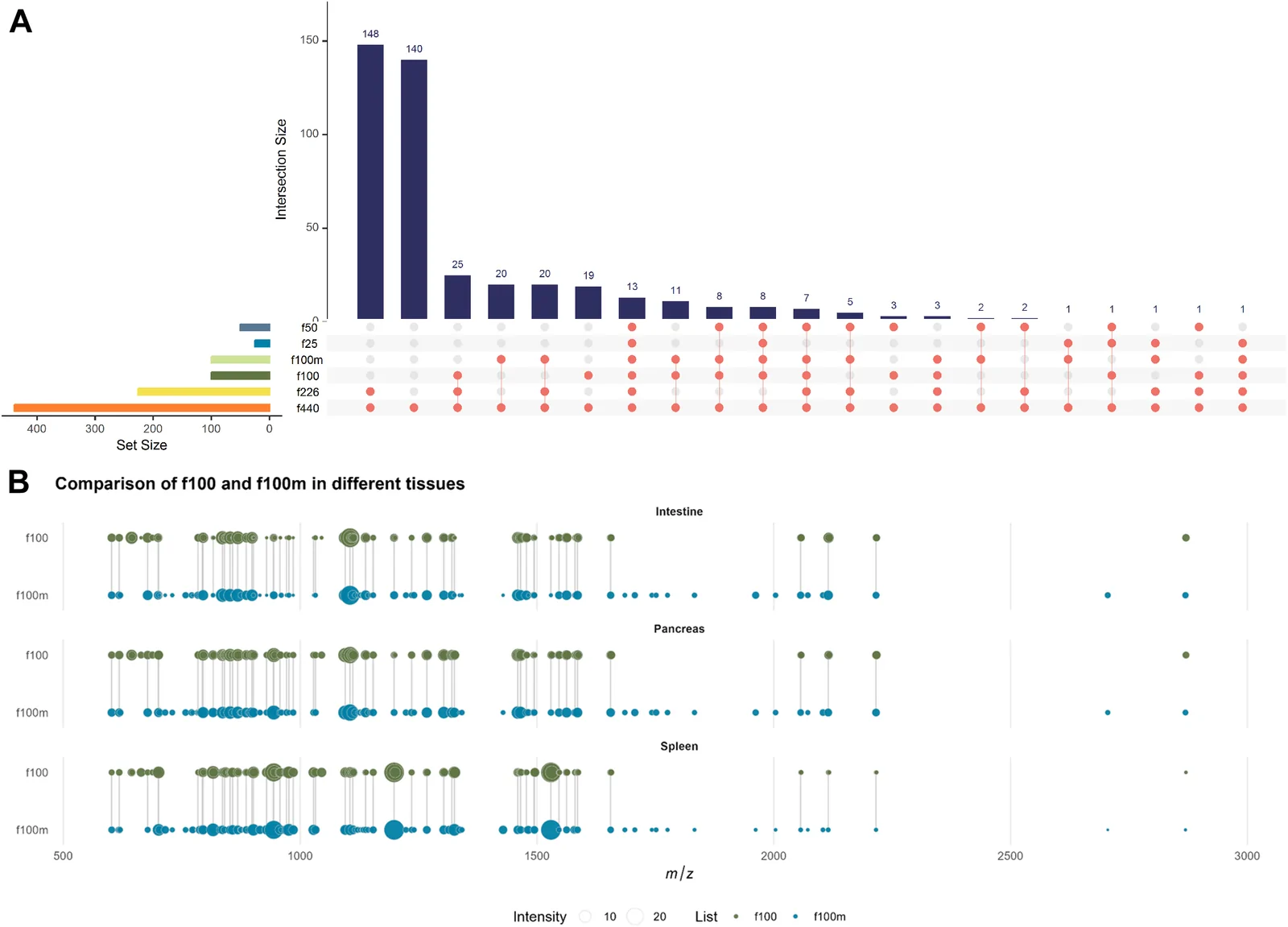
**Comparison of the features in the different selection approaches.***A*, shared features between the different feature selection approaches. *B*, comparison between f100 (features selected by intensity) and f100m (manually selected features to represent to the best of our knowledge the most intense monoisotopic peaks).

Focusing on the f100 features, the average overlap of detected features in Round 1 was approximately 80% across two laboratories and 60% across three laboratories, with variability depending on tissue type. The highest overlap was observed for the intestine (two labs: 93%; three labs: 81%), while the spleen showed the lowest overlap (two labs: 70.3%; three labs: 47%). In Round 2 experiments, the overlap was significantly higher, with an average of over 90% across two laboratories and nearly 80% across three laboratories. Notably, consistently high overlaps were observed across all three tissues.

In addition to the unsupervised classification, we have compared the overall intensity for all features per lab across the different mass lists, the feature reproducibility across the different rounds in the different labs and determined the correlation between the individual datasets.

### Consistency Between Rounds Prioritizes Biological Differences

To evaluate the impact of round or laboratory-specific variations, k-means clustering was applied to the peptide features acquired across three independent experimental rounds conducted in different laboratories. The resulting segmentation maps were used to assess reproducibility of tissue-specific molecular patterns across rounds and to disentangle biological variation from potential batch or laboratory effects (Fig. 2*D*).

When clustering was performed using the full set of detected *m/z* features, spatially coherent clusters emerged across all tissue sections, indicating that the data contain robust biological structure even without filtering. However, comparing replicates across rounds revealed noticeable variations in cluster distribution and spatial boundaries, suggesting that technical differences arising from sample preparation contribute measurably to the clustering outcome. Less significant differences could be associated with the acquisition settings, or instrument-specific factors. Nonetheless, these differences remained secondary to biological organization, major tissue domains remained identifiable, but noise introduced by laboratory conditions reduced cross-round comparability at full feature dimensionality.

To further evaluate sources of variation, PCA was performed on peptide features before and after regression-based adjustment for lab-specific effects (Fig. 4). Both prior to and following correction, samples clustered primarily by tissue type, with intestine, pancreas, and spleen forming distinct groups along the principal components. Differences between laboratories and experimental rounds were minor, as indicated by the scattered distribution associated with the labs and rounds within each tissue cluster. The regression-based adjustment slightly reduced residual lab- and round-specific offsets but preserved the overall tissue-specific structure, confirming that biological variation dominates the observed variance. These findings are also supported by the high concordance between measurement rounds across tissues, with CCC values of 0.78 (spleen), 0.81 (pancreas), and 0.88 (intestine), indicating good to excellent agreement.

**Fig. 4:**
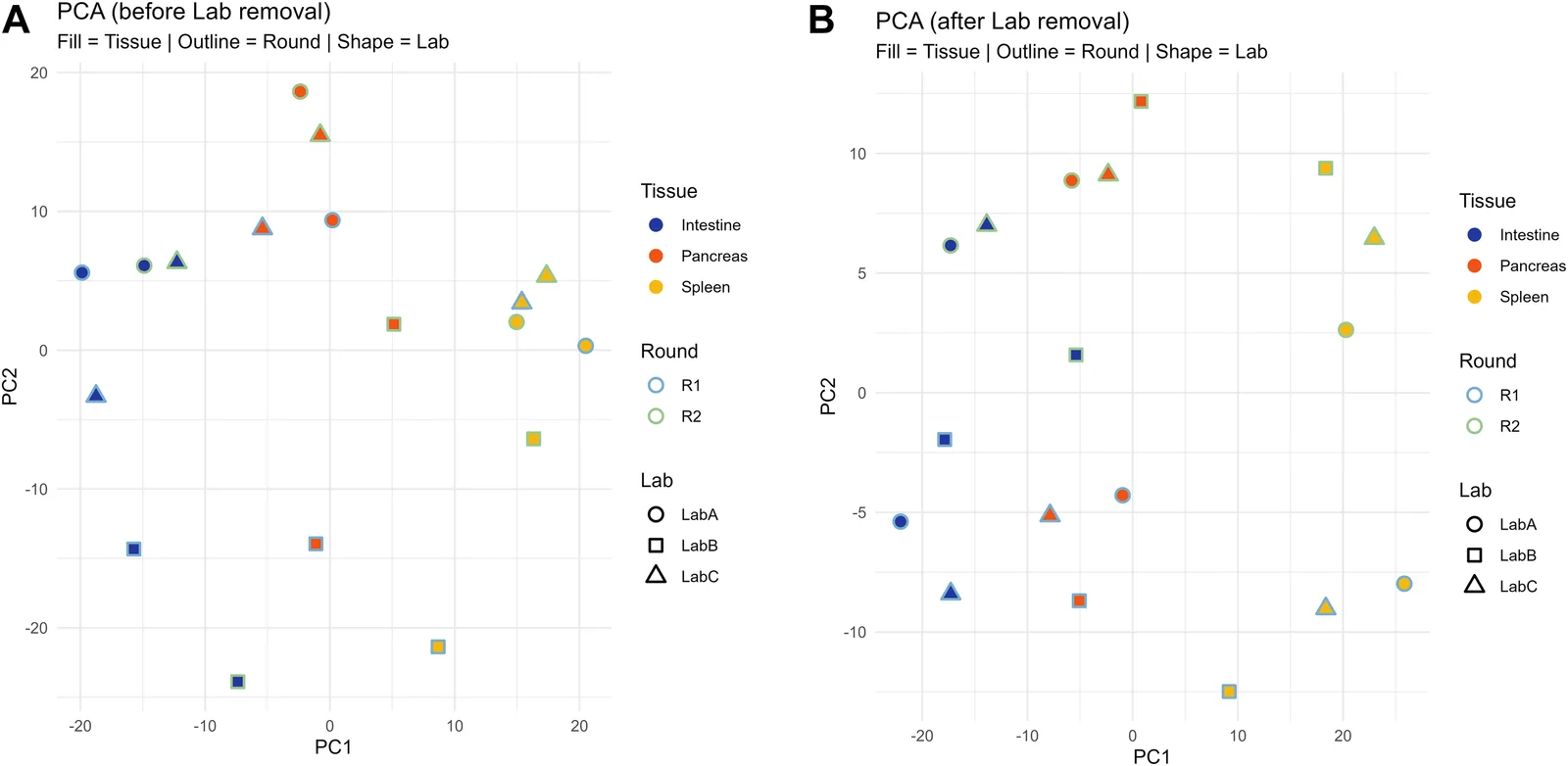
**PCA of MALDI-MSI data before and after batch correction shows tissue-driven separation.** PCA of MSI data (*A*) before and (*B*) after regression-based correction for laboratory batch effects**.** The plots show the first two principal components (PC1 versus PC2) for samples from three tissues (intestine, pancreas, spleen) and two rounds (R1 – centralized data acquisition, R2 – centralized sample preparation). The first two principal components explain 40.2% and 31.9% of the variance before, and 69.9% and 19.9% after regression-based correction. Supplemental Fig. S8, shows the individual effects of the correction on tissue, experimental round and lab with 50% confidence eclipses.

Restricting the analysis to the *m/z* 1150 to 2250 range improved concordance between rounds. In this reduced feature space, the clustering results demonstrated good consistency in the spatial localization and extent of individual clusters. This improvement suggests that limiting the analysis to a biologically relevant mass window mitigates the contribution of less reproducible features that may originate from chemical noise, matrix clusters, or low-abundance signals that fluctuate across instruments and laboratories. The lower *m/z* region (<1000) is inherently more complex, containing a higher proportion of short peptide fragments that are often shared among multiple proteins, as well as increased interference from matrix-related ions. Together, these factors reduce feature specificity and may contribute to lower reproducibility compared with higher-*m/z* peptides. Reducing the analysis to *m/z* 1150 to 2250, technical variability remained detectable but diminished in comparison to the full feature set.

Strong agreement between rounds was observed when the analysis was further reduced to the top 25 most intense features. In this highly selective feature space, clustering patterns were strikingly similar across laboratories, indicating strong preservation of biologically driven spatial structure. Differences attributable to round-specific or laboratory-specific factors became minimal, and the primary drivers of segmentation appeared to be conserved molecular signatures inherent to the tissue rather than technical artifacts. This outcome demonstrates that a small subset of high-confidence, high-intensity features captures the dominant molecular heterogeneity of the tissue while suppressing contributions from noisy or variable signals.

Across all experimental conditions, the spatial distributions of selected peptide ions demonstrate consistent biological patterns (Fig. 5). Collagen alpha-1(XXI) chain (COL21A1) shows robust enrichment in the muscularis region in every dataset (Fig. 5*A*). Tripartite motif-containing protein 28 (TRIM28) likewise localizes predominantly to the muscularis layer of the intestine, with additional pronounced accumulation in vascular structures and in the splenic capsule (Fig. 5*B*). Also, when looking more in detail into these regions (splenic capsule and intestinal muscularis), commonly clustered together, using Receiver–Operating Characteristic Area Under the Curve analysis, it is possible to identify features that confidently differentiate between the regions (Supplemental Table S5).

**Fig. 5:**
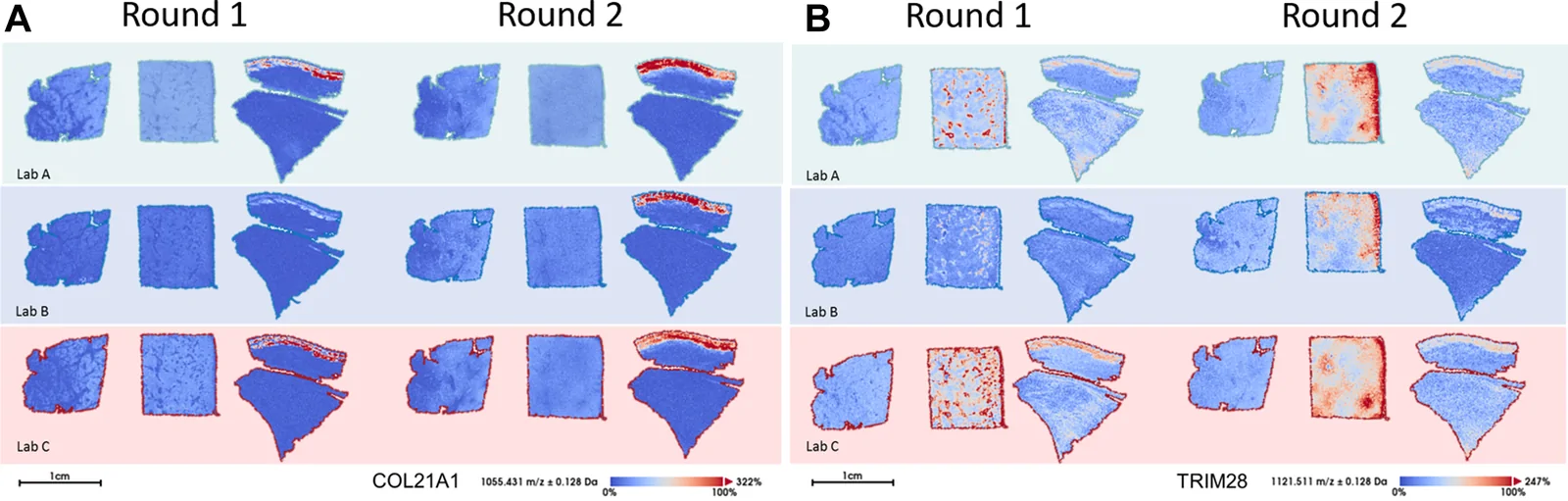
**Spatial distributions of COL21A1 and TRIM28 peptides are reproducible across laboratories and experimental rounds.** Ion distribution of (*A*) *m/z* 1065.4, identified as a peptide fragment of COL21A1 and (*B*) *m/z* 1121.5 identified as a peptide fragment of TRIM28 across different labs and experimental rounds. Round 1: decentralized sample preparation with centralized acquisition. Round 2: centralized sample preparation with decentralized acquisition.

Taken together, these analyses indicate that biological variation consistently outweighs technical variation across all feature selection approaches. While laboratory-specific effects are detectable, particularly when all features are included, they do not obscure the underlying biological organization of the tissue. Moreover, the application of feature selection, particularly selection of high-intensity or biologically enriched features, substantially improves reproducibility between experimental rounds and mitigates laboratory effects. These findings support the robustness of spatial proteomic clustering under multi-site acquisition conditions and underscore the utility of informed feature selection to enhance cross-experiment comparability.

To assess the relative contributions of biological and technical factors to feature variability across datasets, we computed effect sizes (η^2^) for tissue, lab, and round using ANOVA for each feature list (Fig. 6). The resulting η^2^ distributions revealed pronounced dataset-specific patterns. In f100, f100m, f440, and f50, the η^2^ densities for tissue were consistently shifted toward higher values compared with lab and round, indicating that tissue type accounted for a substantial proportion of the variance across many features. Notably, f100m exhibited the strongest tissue-driven separation, with a pronounced right-tail of η^2^ values approaching ∼0.8. f50 showed a similar (though slightly more moderate) right-shift of tissue η^2^ (exceeding lab and round) consistent with a robust biological signal preserved at this feature depth. In f25, tissue also emerged as the primary contributor to variance, although the separation from lab and round was less pronounced than in f100m and somewhat closer to the pattern observed in f50. In contrast, f226 displayed η^2^ distributions for all three factors tightly centered near zero, suggesting minimal variance attributable to either biological or technical sources. Across datasets, lab and round effects were generally small relative to tissue, though moderate overlap between distributions indicated that certain features remained partially influenced by acquisition-specific factors.

**Fig. 6:**
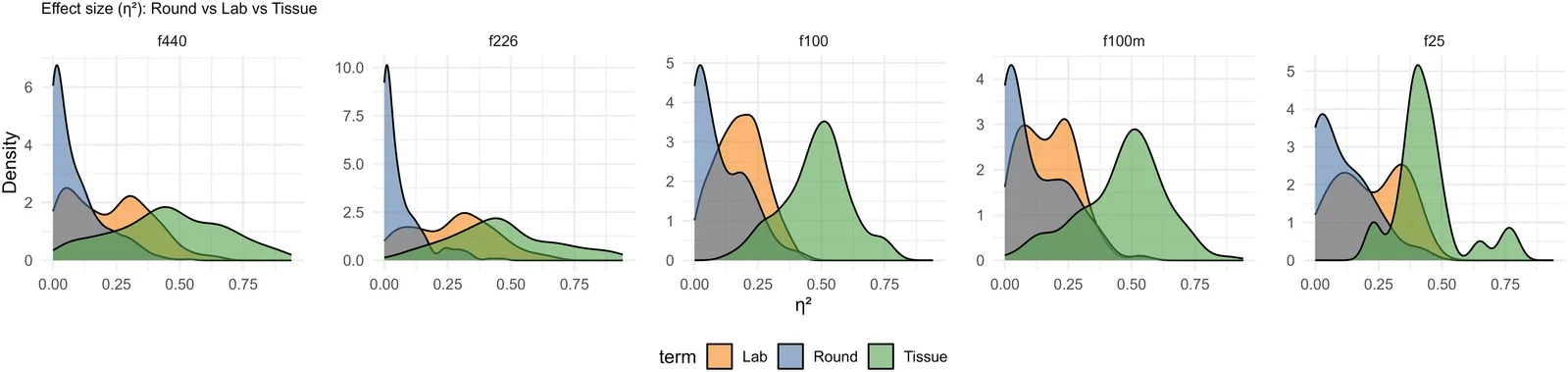
**Effect size (η^2^) distributions from one-way ANOVA models (intensity ∼ factor), computed per feature across datasets (f440, f226, f100, f100m, f25; *left* to *right*).** For each feature, three separate ANOVA models were fitted to quantify variance explained by: experimental round (*blue*; Round 1: centralized data acquisition versus Round 2: centralized sample preparation, representing inter-experimental/batch effects), laboratory (*orange*; three participating laboratories in a ring trial, representing inter-laboratory variation across all tissues and rounds), and tissue type (*green*; three biological tissues: spleen, pancreas, and intestine). Kernel density estimates show the distribution of per-feature η^2^ values for each factor.

### Intralaboratory Variability Remains Low

Although more pronounced differences were detected between measurements in Round 1, intra-laboratory variability remains minimal. Minor intensity differences are observed; however, overall feature expression patterns remain largely consistent. In Round 3, it becomes evident that biological variability continues to outweigh these minor technical fluctuations. This observation is further supported by the correlation analysis (Supplemental Fig. S9), which compares individual tissues measured within the same laboratory. The strongest correlations are consistently observed between tissue sections originating from the same specimen, rather than between measurements acquired at different times. Likewise, analysis of the top 100 most intense features for each tissue type (Supplemental Fig. S9*C*) shows that the predominant features are largely shared, and those exhibiting higher expression within a given tissue type remain stable across measurements.

## Discussion

Reproducibility is crucial for establishing new technologies, particularly when the underlying goal is clinical integration (39, 40). This encompasses not only technical reproducibility but also reproducibility of data analysis (41, 42). Regardless of the outcome, it must be consistent across experiments and settings.

Until now, two multi-institutional studies on reproducibility of characterizing tryptic peptides by MALDI-MSI have been conducted (18, 19). In a report by Buck *et al*., the reproducibility of MALDI-MSI was determined across four laboratories using FFPE multi-organ tissue microarrays (TMAs) (18). Both peptide and metabolite profiles of eight distinct tissue types were acquired and compared for inter- and intra-center variability. The researchers found that inter-center variation for peptides was significantly lower than intra-center variation, whereas metabolites exhibited higher inter-center variability (18). Nonetheless, when focusing on relative univariate signals with discriminatory power, metabolites proved more reproducible across centers than peptides. In terms of absolute signal intensities, multivariate classifiers trained on sufficiently diverse cross-center data achieved average accuracies exceeding 90% for peptides and over 80% for metabolites. These findings show that, even with the common preanalytical complexity of FFPE samples, MALDI-MSI data can be highly reproducible across laboratories, with peptide-based classifiers showing remarkable cross-site transferability. To assess site-to-site reproducibility, Ly *et al*. analyzed the proteomic content of various tissue types, after tryptic digestion, including a tissue microarray (TMA) (19) comprising tumor entities collected from three different centers. The study demonstrated that spatial resolution and data quality are strongly influenced by both instrumentation parameters (*e.g*., laser diameter, spot-to-spot distance) and sample preparation factors (*e.g*., matrix and trypsin application, matrix homogeneity). Importantly, the authors provided evidence that high-speed, high-resolution MALDI–MSI can yield reproducible results across sites when protocols are carefully harmonized.

To evaluate cross-site reproducibility and classifier transferability for MALDI-MSI-based tissue phenotyping, Deininger *et al*. specifically investigated the robustness of the approach against technical variability (20). The study analyzed tryptic peptide signals in a TMA derived from patient samples. After protocol harmonization, classification accuracy reached 78.6%, compared to 67.0% with a non-harmonized protocol and just 36.1% with a mistuned instrument. The authors introduced an optimized data preprocessing pipeline, which substantially improved classification accuracies to 94.3%, 87.3%, and 81.3%, respectively. These findings highlight two key insights: technical variability, such as differences in sample preparation and instrument tuning, significantly compromises classifier generalizability, and carefully designed preprocessing workflows can effectively mitigate site-specific artifacts while preserving biologically relevant signals.

Also addressing data analysis, a study by Fagerer *et al*. aimed to establish a standardized framework for MALDI-MSI data processing through a cross-laboratory comparison involving five different sites (21). The study addresssed key reproducibility challenges inherent to multicenter studies and demonstrated that standardized sample preparation is essential for achieving consistent results across locations.

Our inter-laboratory experiments aimed at evaluating the reproducibility and robustness of the MALDI-MSI approach for peptide/protein analysis in patient derived tissue. In experimental Round 1, samples were prepared independently in each laboratory and analyzed centrally at a single site. In Round 2, all samples were prepared at one laboratory and distributed to multiple sites for data acquisition on respective rapifleX MALDI-TOF instruments. Round 3, evaluated intra-laboratory reproducibility, with samples prepared and measured at a single location at different time points.

Unsupervised analysis of the data, utilizing all detected features (total of 440 features), obtained by feature finding in SCiLS Lab Pro, revealed greater reproducibility when all samples were prepared by a single individual in one lab. In contrast, differences between instruments had a comparatively smaller impact on reproducibility. These findings suggest that, even when standardized protocols are followed, certain sources of variability, particularly in sample preparation and laboratory environmental conditions (*e.g*., temperature and humidity), can significantly influence the measurement outcome. This effect is likely to be further amplified by the high sensitivity of modern instruments, where even minor deviations can affect results. Nevertheless, high sensitivity is indispensable for detecting subtle differences within complex samples, such as patient derived tissue sections.

Environmental factors such as humidity and external temperature are well known to affect sample preparation and measurement outcomes, but they remain difficult to fully control or correct for in routine experimental workflows (43). Therefore, robust data preprocessing and analysis pipelines are necessary to account for and mitigate these subtle experimental variabilities. Importantly, the variability observed across experimental rounds predominantly reflects technical rather than biological variation, as identical tissue sections were analyzed. This distinction is critical when interpreting analyses where technical variance can otherwise be misattributed to biological heterogeneity.

One critical step that significantly impacts the outcome of data analysis is peak or feature selection (44). To improve this process, we explored several strategies for optimizing feature selection (36, 45). One commonly used approach involves selecting the most abundant peaks within the acquired mass range. Using this method, we selected the top 100 *m/z* values with the highest relative intensities. While being the easiest one to implement, it often leads to the selection of molecular ions from the same peptide fragment potential challenging proceeding data analysis. Moreover, we observed that most of these features fell within a specific mass range, approximately *m/z* 600 to 1500. Peaks in this range often include peptides derived from trypsin autolysis or clusters of matrix, which can overshadow peaks of biological interest. Despite its limitations, this approach inspired us to refine our strategy by focusing on specific mass ranges.

To address this, we selected the mass range of *m/z* 1150 to 2250, as the range of *m/z* 600 to 800 showed greater variability in peak intensity (Supplemental Fig. S2*C*) and the range of *m/z* 600 to 1150 exhibited a higher number of high-intensity background peaks. Above *m/z* 2250, the signal-to-noise ratio decreases, and peptide identification becomes more challenging. All features detected in the *m/z* 1150 to 2250 range, a total of 226 features, were considered. Within this mass range, the data showed the very good spatial correlation and the least variability in peak intensity per lab per round. However, by employing this approach we are discarding almost half (48%) of the acquired data at the lower and higher mass ranges, which still include highly meaningful features for biological interpretation. Therefore, we devised a third approach that aimed at including features across the whole acquisition range and that also considered the variability of the noise signal across different mass ranges. This strategy involved dividing the mass spectrum into multiple ranges and selecting features proportionally to their representation in the original dataset. For example, if 20% of the features in the initial dataset were within the mass range of *m/z* 2000 to 2400, 20% of the selected features were also included in this feature list. Using this approach, we selected the top 25 *m/z* features (Supplemental Fig. S2*A* and Supplemental Table S4) across the entire mass range while preserving the original data ratio. While using merely 25 features may seem too reductive, when looking at average intensity distribution there is a good representation of the whole dataset. Moreover, the k-means clustering (Fig. 2*D*) yielded very good comparability between the different datasets. However, carrying out this type of feature selection method can be time consuming, and depending on the sample, different mass ranges need to be considered, leading to challenges when comparing different datasets.

Another commonly employed feature selection strategy involves manual selection of monoisotopic peaks followed by intensity-based filtering. Although widely used, this approach is very time-consuming and relies on substantial prior knowledge for accurate peak selection; moreover, distortions of isotopic patterns may still render it prone to errors. Nevertheless, when directly compared with the f100 approach (Fig. 3*B*), this method captures a larger number of detectable analytes, especially in the *m/z* 1200 to 2000 (Supplemental Fig. S9*A* and Supplemental Table S4). As expected, the f100m approach yields more reproducible outcomes in unsupervised analyses when compared to the f100.

Effect size analysis (Fig. 6) showed that most datasets capture biologically meaningful, tissue-specific variation, whereas f226 exhibited poorly separated η^2^ values for tissue, round, and lab, consistent with a low-informative or noisy feature profile. Effect size patterns revealed marked differences in tissue signal strength across acquisition modes, with important consequences for biological interpretation and reproducibility. A pronounced rightward shift of tissue η^2^ in f100, f100m, f440, and f50, and to a lesser extent in f25, indicates robust preservation of biochemical tissue distinctions. The particularly strong tissue effect observed in f100m suggests enhanced contrast between biological groups, potentially reflecting improved sensitivity, dynamic range, or ion fragmentation behavior. In contrast, the near-zero η^2^ values in f226 indicate minimal structured variance, biological or technical, likely limiting its suitability for biological inference. Overall, the predominance of tissue-driven variance across most datasets supports strong biological reproducibility across sites and acquisition rounds, while the underperformance of f226 highlights the need for dataset-specific quality assessment in multi-center MSI studies.

Taken together, our results indicate that feature selection strategies should be tailored to the experimental objective and sample type. At the same time, they underscore the importance of robust and standardized feature finding and selection approaches that promote comparability across datasets. Such strategies are essential to ensure analytical robustness and reproducibility, particularly in multi-center studies and in translational or clinical contexts where consistency across acquisition rounds and data processing are critical. Approaches that incorporate features across the full mass range, while explicitly accounting for noise and intensity variability, may therefore provide a balanced compromise between data reduction, biological representativeness, and cross-dataset comparability. Nevertheless, several limitations of this study should be acknowledged. The experiments were restricted to a single matrix and digestion workflow and were conducted on a single MALDI-TOF platform, which may limit generalization to other MSI modalities or instrument types. Furthermore, the analysis focused on feature-level comparisons rather than peptide identification and thus does not capture potential variability at the level of protein inference.

More broadly, while a range of analytical strategies can be applied to mass spectrometry imaging data, the choice of feature list remains a critical determinant of robustness and susceptibility to random error. The absence of standardized guidelines for spatial data analysis and reporting further hampers meaningful comparison across studies. Although we do not propose a single optimal feature selection strategy, our results systematically demonstrate that variations in feature selection can substantially affect reproducibility and biological interpretation. These observations reinforce the need for increased standardization of data processing workflows within the community to enhance robustness and cross-study comparability. As MALDI-MSI advances toward clinical and translational applications, systematic evaluation and transparent reporting of analytical choices will be essential to ensure that observed spatial patterns are reproducible, biologically meaningful, and transferable across laboratories.

## Data availability

Raw data are available via ProteomeXchange with identifier PXD076829. Protein identification and quantification results from the database proteomics search are also available in the project folder.

## Supplemental Data

This article contains supplemental data.

## Declaration of Generative AI and AI-Assisted Technologies in the Writing Process

The authors used Grammarly to assist with language polishing and correction of grammatical errors in the manuscript. All text was reviewed and approved by the authors, who take full responsibility for the content of this work.

## Conflict of Interest

The authors declare the following financial interests/personal relationships which may be considered as potential competing interests. J. P. L. G. has served as speaker for Bruker and BI. M.G. has served as speaker for Bruker. C. H. has served as speaker for- and is co-hosting scientific events with Bruker. O. K. has served as speaker for Bruker. K. S. has attended Advisory Boards and served as speaker for Roche, BMS, MSD, Merck, Bruker and BI. All other authors declare that no conflicts of interest exist.
