# Supplementary material for: Multi-Center Study on Sample Preparation and Feature Selection Effects in Reproducible Mass Spectrometry Imaging for Clinical Translation
Source: Mol Cell Proteomics. 2026 Sep 10;25(10):101658. doi: 10.1016/j.mcpro.2026.101658 (PMC13697253; doi:10.1016/j.mcpro.2026.101658)
Supplement: Supplementary file 1 [file mmc1.docx]

**Multi-Center Study on Sample Preparation and Feature Selection Effects in Reproducible Mass Spectrometry Imaging for Clinical Translation**

Juliana P. L. Gonçalves ^1, †^, Marta Grzeski ^2, †^, Gentiane Krasniqi^3^, Carsten Hopf^3^, Oliver Klein^2, *^, Kristina Schwamborn^1, *^

^1^Institute of Pathology, School of Medicine and Health, Technical University of Munich, Trogerstraße 18, 81675 Munich, Germany

^2^Imaging Mass Spectrometry Unit, Berlin Institute of Health (BIH) at Charité, Augustenburger Platz 1, 13353 Berlin, Germany

^3^Center for Mass Spectrometry and Optical Spectroscopy (CeMOS), Technische Hochschule Mannheim, Paul-Wittsack-Str. 10, 68163 Mannheim, Germany

†,* These authors contributed equally to this work

**Supplemental Information**

**Table S1.** Sample preparation specifications across the different labs

| **Step** | **Settings/ Device/ Units** | **TUM** | **CeMOS** | **BIH** |
| --- | --- | --- | --- | --- |
| FFPE slide drying (after sectioning) | 37°C oven | 🗸 | 🗸 | 🗸 |
| FFPE slide heated (before measurement) | 80°C oven | 15 min | 15 min | 15 min at 75°C + 10 min at 80-85°C |
| First Xylene bath | Room temp | 🗸 | 🗸 | 🗸 |
| Second Xylene bath | Room temp | 🗸 | 🗸 | 🗸 |
| 100% Isopropanol | Room temp | 🗸 | 🗸 | 🗸 |
| 100% EtOH | Room temp | 🗸 | 🗸 | 🗸 |
| 95% EtOH/deionised water | Room temp | 🗸 | 🗸 | 🗸 |
| 70% EtOH/de-ionised water | Room temp | 🗸 | 🗸 | 🗸 |
| 50% v/v EtOH/de-ionised water | Room temp | 🗸 | 🗸 | 🗸 |
| De-ionised water | Room temp | 🗸 | 🗸 | 🗸 |
| Antigen retrieval device | BioCare Decloaking Chamber | 🗸 | 🗸 | Steamer |
| Antigen retrieval buffer | De-ionised water | 🗸 | 🗸 | 🗸 |
| Antigen retrieval temperature | 110°C ± 2°C | 🗸 | 🗸 | 98°C ± 2°C |
| Antigen retrieval duration | 20 min | 🗸 | 🗸 | 🗸 |
| Cooling | Room temperature water exchanges | 3 | 3 | 0 |
| Drying | Desiccator | 15 min | 10 min | 15 min |
| Trypsin | batch number | 560791 | 542882 | 424100 |
| Spraying device |  | HTX TM | HTX M3 | HTX TM |
| Nozzle temperature | 30°C | 🗸 |  | 🗸 |
| Enzyme distribution | µg/mm^2^ | 0.0048 | 0.004303 | 0.00430 |
| N_2_ flow pressure | psi | 10 | 10 | 11 |
| Digestion temperature | 50°C | 🗸 | 🗸 | 🗸 |
| Shots | 500 shots | 🗸 | 🗸 | 🗸 |
| Frequency | 10kHz | 🗸 | 🗸 | 🗸 |
| Laser application (Sample carrier tab) | Imaging 50 | 🗸 | 🗸 | 🗸 |
| Smartbeam | M5 small | 🗸 | 🗸 | 🗸 |
| Scan range | 11,11 | 🗸 | 🗸 | 🗸 |
| Resulting field size | 50, 50 | 🗸 | 🗸 | 🗸 |
| Detection range | 600-3200 | 🗸 | 🗸 | 🗸 |
| Global Offset Attenuator |  | 2% | 5% | 0.80% |

**Table S2.** Site specific chemicals and solvents

| **Chemical/ Product** | **Supplier TUM** | **Supplier BIH** | **Supplier CeMOS** |
| --- | --- | --- | --- |
| Xylene | Carl-Roth, 4436 | Thermo Scientific, 140990025 | Sigma Aldrich/Merck, 1.08633 |
| Isopropanol | Carl-Roth, 0733 | Merck 1.02781 | VWR, 84881.320 |
| Ethanol | Carl-Roth, 5054 | Sigma Aldrich/Merck, 1.00986 | VWR, 20821.330 |
| Potassium sulfate | Carl-Roth, P022 | Fisher Scientific, P/6960/53 | Carl-Roth, CN79.1 |
| Ammonium bicarbonate | Sigma-Aldrich/ Merck, 09830 | Sigma Aldrich, 09830 | AppliChem, A3689 |
| Glycerol | Sigma-Aldrich/ Merck, G9012 | Merck, 1.04094 | Carl-Roth, 6962.1 |
| Trypsin | Promega, V5111 | Promega, V5111 | Promega, V5111 |
| Acetonitrile | Carl-Roth, 8824 | Carl Roth, HN40.2 | VWR, 83640.320 |
| Digestion chamber | Emsa 508570 Clip & Close | Emsa 508570 Clip & Close | Emsa 508570 Clip & Close |
| HCCA | Sigma-Aldrich/ Merck, 70990 | Bruker, 8201344 | Bruker, 8201344 |
| TFA | Carl-Roth, P088 | Merck, 108262 | Merck, 108262 |

Table S3. Acquisition details for each measurement included in this study.

|  | Round 1 | | | Round 2 | | | Round 3 | |
| --- | --- | --- | --- | --- | --- | --- | --- | --- |
| Measurement | **1.1** | **1.2** | **1.3** | **2.1** | **2.2** | **2.3** | **3.1** | **3.2** |
| Laser Power | 55 | 70 | 46 | 55 | 55 | 50 | 55 | 52 |
| Detector Gain | 32.64 | 1.12 | 1 | 41.9 | 1.12 | 1 | 1.12 | 1.12 |
| Sample Rate | 1.25 | 1.25 | 1.25 | 1.25 | 1.25 | 1.25 | 1.25 | 1.25 |
| PIE Voltage | 2610 | 2660 | 2610 | 2617 | 2610 | 2660 | 2660 | 2660 |
| Reflector 1 | 20950 | 20800 | 20950 | 20820 | 20950 | 20800 | 20800 | 20800 |
| Reflector 2 | 1085 | 1085 | 1085 | 1085 | 1085 | 1085 | 1085 | 1085 |
| Reflector 3 | 8850 | 8700 | 8850 | 8590 | 8850 | 8700 | 8700 | 8700 |

**Figure S1.** Detector calibration spectra for round 2 (centralized sample preparation, decentralized data acquisition). Peptide calibration standard II composed of Bradykinin Fragment 1-7, Angiotensin II, Angiotensin I, Substance P, Bombesin, Renin Substrate, ACTH clip 1-17, ACTH clip 18-39, and Somatostatin 28, was utilized for calibration.

**Figure S2**. Data distribution analysis across the mass range.

**Figure S3.** PCA, component 1 and 2 explain 55.8% of the data; components 1, 2 and 3 explain 62.9% of the data. Pink region corresponds to the region created for general feature selection, intersecting areas of all tissues.

**Figure S4.** PCA of samples across six datasets**.** PC1 vs. PC2 score plots are shown for each dataset (f25, f50, f100, f100m, f226, f440); axis labels report the percentage of variance explained. Panels (a–f) are colored by Lab (LabA, LabB, LabC), and panels (g–l) are colored by Round (R1, R2). PCA was performed per dataset on centered, non‑scaled feature matrices with missing intensities set to zero. The paired layouts enable direct visual comparison of laboratory versus round effects within each dataset.

**Table S4.** *m/z* features in each reduced feature list

| **f100** | | **f100m** | | **f25** | **f50** |
| --- | --- | --- | --- | --- | --- |
| 602.3 | 1106.56 | 602.3 | 1115.5 | 644.34 | 602.28 |
| 618.29 | 1107.53 | 616.33 | 1116.48 | 795.35 | 618.26 |
| 644.34 | 1111.54 | 618.29 | 1121.51 | 836.4 | 644.31 |
| 645.31 | 1112.57 | 619.31 | 1127.52 | 852.38 | 678.31 |
| 664.33 | 1138.52 | 620.29 | 1131.53 | 868.4 | 688.32 |
| 678.31 | 1139.55 | 678.31 | 1133.49 | 886.42 | 700.35 |
| 688.32 | 1154.55 | 700.35 | 1138.52 | 898.49 | 758.36 |
| 700.35 | 1198.64 | 701.37 | 1143.51 | 944.46 | 785.37 |
| 701.37 | 1199.66 | 703.33 | 1149.52 | 1095.53 | 795.36 |
| 784.41 | 1200.64 | 715.35 | 1154.55 | 1105.51 | 816.43 |
| 785.34 | 1235.59 | 730.31 | 1198.64 | 1111.55 | 836.42 |
| 793.46 | 1267.65 | 758.36 | 1223.56 | 1138.53 | 840.43 |
| 795.36 | 1268.63 | 771.36 | 1235.59 | 1198.62 | 842.45 |
| 796.39 | 1303.57 | 773.37 | 1240.62 | 1267.62 | 852.4 |
| 816.38 | 1304.6 | 784.41 | 1267.65 | 1303.56 | 868.41 |
| 817.41 | 1320.63 | 786.37 | 1303.57 | 1320.65 | 874.4 |
| 836.42 | 1321.66 | 788.42 | 1314.62 | 1459.69 | 886.42 |
| 837.4 | 1325.71 | 793.46 | 1320.63 | 1465.68 | 898.47 |
| 840.43 | 1326.69 | 795.36 | 1325.71 | 1477.75 | 901.47 |
| 841.45 | 1459.68 | 815.4 | 1336.61 | 1562.78 | 943.51 |
| 842.48 | 1460.66 | 816.38 | 1341.6 | 1585.77 | 944.48 |
| 843.46 | 1461.69 | 831.43 | 1428.7 | 2104.06 | 1095.53 |
| 852.4 | 1465.7 | 836.42 | 1459.68 | 2115.11 | 1105.53 |
| 853.38 | 1466.67 | 840.43 | 1465.7 | 2216.06 | 1111.57 |
| 857.44 | 1477.72 | 852.4 | 1477.72 | 2869.39 | 1116.48 |
| 868.43 | 1478.75 | 857.44 | 1481.82 |  | 1127.52 |
| 869.41 | 1493.75 | 868.43 | 1493.75 |  | 1133.54 |
| 886.42 | 1494.73 | 874.4 | 1494.73 |  | 1138.52 |
| 887.4 | 1495.75 | 875.47 | 1529.72 |  | 1154.55 |
| 898.49 | 1529.72 | 882.46 | 1546.78 |  | 1198.69 |
| 899.47 | 1530.7 | 886.42 | 1562.76 |  | 1235.59 |
| 901.47 | 1531.73 | 890.38 | 1578.75 |  | 1267.65 |
| 902.45 | 1546.78 | 898.49 | 1580.75 |  | 1303.57 |
| 929.48 | 1547.76 | 901.47 | 1585.73 |  | 1320.63 |
| 943.51 | 1562.76 | 914.42 | 1655.77 |  | 1325.71 |
| 944.48 | 1563.79 | 915.45 | 1685.78 |  | 1459.68 |
| 945.46 | 1580.75 | 929.48 | 1706.8 |  | 1465.67 |
| 946.49 | 1585.73 | 943.51 | 1742.77 |  | 1477.72 |
| 957.48 | 1586.76 | 944.48 | 1751.81 |  | 1529.72 |
| 958.51 | 1587.74 | 957.48 | 1775.86 |  | 1546.78 |
| 971.51 | 1655.77 | 960.42 | 1832.85 |  | 1562.79 |
| 976.4 | 1656.8 | 971.51 | 1961.93 |  | 1580.75 |
| 985.54 | 2056.95 | 976.4 | 2003.97 |  | 1585.76 |
| 1028.55 | 2057.97 | 985.54 | 2056.95 |  | 1655.8 |
| 1032.56 | 2115.11 | 1028.55 | 2072.05 |  | 1706.8 |
| 1045.51 | 2116.09 | 1032.56 | 2104.06 |  | 1834.9 |
| 1094.53 | 2117.11 | 1093.51 | 2115.11 |  | 1923.91 |
| 1095.51 | 2216.09 | 1095.51 | 2216.09 |  | 1961.98 |
| 1096.54 | 2217.06 | 1105.53 | 2705.29 |  | 2057.95 |
| 1105.53 | 2870.44 | 1111.54 | 2869.41 |  | 2115.11 |

**Figure S5.** Spectra of intestine tissue sections from the different labs and different rounds

**Figure S6**. Spectra of pancreas tissue sections from the different labs and different rounds

**Figure S7.** Spectra of spleen tissue sections from the different labs and different rounds

**Figure S8**. PCA before and after correction for laboratory effects. PCA scores are shown for the first two principal components based on log10-transformed feature intensities. Panels A–C display the uncorrected data, with 50% confidence ellipses for (A) tissue type, (B) experimental round, and (C) laboratory. Panels D–F show the corresponding PCA after regression-based removal of the laboratory effect, again with 50% confidence ellipses for (D) tissue type, (E) experimental round, and (F) laboratory. Points represent individual samples and are colored by the grouping variable indicated in each panel. Ellipses represent group dispersion based on a 50% concentration region under a multivariate normal approximation. Variance explained by the first two principal components is indicated on the axes.

**Figure S9**. Round 3 - Intra-laboratory consistency. (a) Average feature intensity per tissue (Pancreas, Spleen, Stomach; consistent colors across panels) for each round, shown as bar plots across *m/z* features (facets are separated by measurement timeline, matching Round 1 (top) and Round 2 (bottom)). (b) Unsupervised spatial segmentations of representative sections for the two rounds, (I) Round 1 and (II) Round 2, illustrating concordant tissue‑like regions (colors match tissue labels). (c) Pearson correlation heatmap of tissue‑wise mean spectra across rounds; strong within‑tissue, across‑round correlations indicate high reproducibility, whereas lower off‑diagonals reflect tissue specificity. (d) Tissue‑specific intensity profiles comparing Round 1 vs. Round 2 (lines), showing conservation of major peaks across rounds for each tissue. (e) Composition of tissue classes (stacked bars) by round for different feature sets (440, 226, 100, 25 features), summarizing how tissue proportions are preserved as the number of retained features decreases.

**Table S5.** ROC-AUC analysis of the muscularis vs capsule regions. The regions were digested following the same protocol as for MSI measurements followed by extraction and identification of the *m/z* with the highest AUC-ROC by nLC-ESI-MS/MS.

| **Muscularis_vs_capsule MALDI [M+H]** | **AUC** | **ESI [M+H]** | **ESI [M]** | **Peptide ID Score** | **Protein coverage [%]** | **Accession** | **Potential protein** |
| --- | --- | --- | --- | --- | --- | --- | --- |
| 767.454 | 0.99880188 | na |  |  |  |  | no identification |
| 822.488 | 0.99725228 | na |  |  |  |  | no identification |
| 875.519 | 0.99662137 | na |  |  |  |  | no identification |
| 976.49 | 0.98138318 | 976.45 | 975.4410 | 54.24 | 73.00 | P60709\|ACTB_HUMAN | Actin (peptide not unique) |
| 1032.606 | 0.99887347 | 1032.54 | 1031.5359 | 52.58 | 80.00 | P17661\|DESM_HUMAN | Desmin |
|  |  | 1032.56 | 1031.5499 | 49.10 | 52.61 | P55072\|TERA_HUMAN | Transitional endoplasmic reticulum ATPase |
| 1055.577 | 0.99998745 | 1055.52 | 1054.5083 | 34.58 | 4.91 | Q96P44\|COLA1_HUMAN | Collagen type XXI alpha 1 chain |
| 1121.609 | 0.0299896 | 1121.53 | 1120.5182 | 51.82 | 38.68 | Q13263\|TIF1B_HUMAN | Transcription intermediary factor 1-beta |
|  |  | 1121.55 | 1120.5400 | 46.89 | 27.74 | P52209\|6PGD_HUMAN | 6-phosphogluconate dehydrogenase |
| 1198.73 | 0.9701769 | 1198.67 | 1197.6605 | 55.10 | 44.00 | P16402\|H13_HUMAN | Histone H1.2/ H1.3/ H1.4 (peptide not unique) |
|  |  | 1198.71 | 1197.6982 | 55.17 | 73.00 | P60709\|ACTB_HUMAN | Actin (peptide not unique) |
| 1209.634 | 0.99584986 | 1209.55 | 1208.5417 | 54.96 | 49.90 | P35749\|MYH11_HUMAN | Myosin-11 |
| 1478.794 | 0.16177248 | 1477.71 | 1476.7065 | 55.22 | 58.55 | P18206\|VINC_HUMAN | Vinculin |
|  |  | 1477.75 | 1476.7435 | 55.37 | 34.71 | Q92841\|DDX17_HUMAN | Probable ATP-dependent RNA helicase DDX17 |
| 1501.765 | 0.99909948 | 1501.73 | 1500.7208 | 53.51 | 48.87 | Q13813\|SPTN1_HUMAN | Spectrin alpha chain |
|  |  | 1501.73 | 1500.7208 | 54.84 | 71.88 | P62736\|ACTA_HUMAN | Actin (peptide not unique) |
|  |  | 1501.74 | 1500.7356 | 55.37 | 29.32 | Q9NUQ9\|CYRIB_HUMAN | Protein FAM49B (peptide not unique) |
|  |  | 1501.74 | 1500.7356 | 55.37 | 20.74 | Q9H0Q0\|CYRIA_HUMAN | Protein FAM49A (peptide not unique) |
| 1950.984 | 0.99970272 | 1950.97 | 1949.9595 | 55.40 | 32.96 | P12111\|CO6A3_HUMAN | Collagen alpha-3(VI) chain |
|  |  | 1950.99 | 1949.9847 | 55.40 | 20.20 | Q6UN15\|FIP1_HUMAN | Pre-mRNA 3'-end-processing factor FIP1 |
